# Comparative Analysis of Temperature Rise between Convective Heat Transfer Method and Computational Fluid Dynamics Method in an Anatomy-Based Left Atrium Model during Pulsed Field Ablation: A Computational Study

**DOI:** 10.3390/jcdd10020056

**Published:** 2023-01-30

**Authors:** Lianru Zang, Kaihao Gu, Xingkai Ji, Hao Zhang, Shengjie Yan, Xiaomei Wu

**Affiliations:** 1 Center for Biomedical Engineering, School of Information Science and Technology, Fudan University, Shanghai 200438, China; 2 Academy for Engineering and Technology, Fudan University, Shanghai 200433, China; 3 Key Laboratory of Medical Imaging Computing and Computer-Assisted Intervention (MICCAI) of Shanghai, Fudan University, Shanghai 200032, China; 4 Shanghai Engineering Research Center of Assistive Devices, Shanghai 200093, China; 5 Yiwu Research Institute, Fudan University, Yiwu 322000, China

**Keywords:** pulsed field ablation, atrial fibrillation, pulsatile blood flow, convective heat transfer, computational fluid dynamics, finite element analysis

## Abstract

The non-thermal effects are considered one of the prominent advantages of pulsed field ablation (PFA). However, at higher PFA doses, the temperature rise in the tissue during PFA may exceed the thermal damage threshold, at which time intracardiac pulsatile blood flow plays a crucial role in suppressing this temperature rise. This study aims to compare the effect of heat dissipation of the different methods in simulating the pulsatile blood flow during PFA. This study first constructed an anatomy-based left atrium (LA) model and then applied the convective heat transfer (CHT) method and the computational fluid dynamics (CFD) method to the model, respectively, and the thermal convective coefficients used in the CHT method are 984 (W/m^2^*K) (blood-myocardium interface) and 4372 (W/m^2^*K) (blood–catheter interface), respectively. Then, it compared the effect of the above two methods on the maximum temperature of myocardium and blood, as well as the myocardial ablation volumes caused by irreversible electroporation (IRE) and hyperthermia under different PFA parameters. Compared with the CFD method, the CHT method underestimates the maximum temperature of myocardium and blood; the differences in the maximum temperature of myocardium and blood between the two methods at the end of the last pulse are significant (>1 °C), and the differences in the maximum temperature of blood at the end of the last pulse interval are significant (>1 °C) only at a pulse amplitude greater than 1000 V or pulse number greater than 10. Under the same pulse amplitude and different heat dissipation methods, the IRE ablation volumes are the same. Compared with the CFD method, the CHT method underestimates the hyperthermia ablation volume; the differences in the hyperthermia ablation volume are significant (>1 mm^3^) only at a pulse amplitude greater than 1000 V, a pulse interval of 250 ms, or a pulse number greater than 10. Additionally, the hyperthermia ablation isosurfaces are completely wrapped by the IRE ablation isosurfaces in the myocardium. Thus, during PFA, compared with the CFD method, the CHT method cannot accurately simulate the maximum myocardial temperature; however, except at the above PFA parameters, the CHT method can accurately simulate the maximum blood temperature and the myocardial ablation volume caused by IRE and hyperthermia. Additionally, within the range of the PFA parameters used in this study, the temperature rise during PFA may not lead to the appearance of additional hyperthermia ablation areas beyond the IRE ablation area in the myocardium.

## 1. Introduction

Atrial fibrillation (AF) is one of the most common arrhythmias in the clinic. Aside from medication, left atrium (LA) radiofrequency ablation (RFA) is currently the gold standard for AF clinical treatment, which works by destroying the local myocardium to isolate abnormal electrical signals causing the arrhythmia. However, RFA still has important limitations, exceptionally high recurrence rates, significant complication rates, and lengthy procedure times [1]. Pulsed Field ablation (PFA) is a promising new non-thermal ablation modality based on cell membrane irreversible electroporation (IRE), which is applied to the myocardium and leads to disruptions in the cellular membrane integrity and cell homeostasis, eventually resulting in cell apoptosis and replacement fibrosis. The promise of PFA is the attainment of highly efficient ablation procedures with short procedural times; the creation of continuous, transmural ablations; and an enhanced safety profile with minimal to no collateral damage [2].

One of the prominent advantages of PFA compared with RFA is that the former is a non-thermal ablation method. However, it is not that there is no temperature rise in the tissue during PFA, but PFA non-preferentially causes tissue ablation through hyperthermia. In fact, the temperature rise in the tissue during PFA is directly related to the energy applied to the tissue and the time for which the energy is applied. At higher PFA doses, collateral Joule heating may cause unwanted thermal damage in the tissue [3]. For the PFA in AF treatment, in the circumstances, the mechanism of myocardial ablation caused by PFA changes from IRE alone to IRE combined with hyperthermia, and it may lead to some of the same potentially life-threatening complications during PFA as during RFA: The rise of blood temperature between 50 °C and 80 °C may lead to the formation of soft thrombi, increasing the risk of stroke, and when the temperature exceeds 100 °C, the water in blood or myocardium experiments a phase change from liquid to gas and an audible steam pops (intramyocardial gas formation) may occur, resulting in a myocardial tear or tamponade [4].

Intracardiac pulsatile blood flow plays a crucial role in suppressing this temperature rise. In current computational studies on RFA, the convective heat transfer (CHT) method and the computational fluid dynamics (CFD) method are usually used to simulate the effect of heat dissipation from pulsatile blood flow on the catheter and myocardium. Although the CFD method can more realistically simulate this heat dissipation than the CHT method, the application of the CFD method leads to the model based on the electric-thermal-fluid dynamics coupling, except for the electric-thermal coupling used to calculate the temperature rise in myocardium and blood caused by the current as with the CHT method, the thermal-fluid dynamics coupling is required to calculate the effect of heat dissipation from the pulsatile blood flow. At the same time, if using the anatomy-based rather than the simplified LA model, the model construction process and the computational complexity brought by the CFD method are significantly increased compared with the CHT method. However, in some instances, it is difficult to access adequate software and/or have the expertise necessary to solve the computational problems of such an accurate model based on a triple-coupled problem [5].

In a review of relevant studies, no computational study of PFA in AF treatment has been conducted to compare the effect of the heat dissipation of the different methods in simulating the pulsatile blood flow during PFA. Accordingly, this study used an anatomy-based LA model to compare the effect of the above two methods on the maximum temperature of myocardium and blood, as well as the myocardial ablation volumes caused by irreversible electroporation (IRE) and hyperthermia under different PFA parameters. This study aims to (1) provide a reference for selecting which method to simulate the effect the heat dissipation from pulsatile blood in further studies in order to achieve the optimal choice of obtaining results that are as consistent as possible with actual PFA, or to shorten the model construction process and reduce the computational complexity without compromising the reasonableness of the results and (2) evaluate whether the temperature rises during PFA lead to the tissue adjacent to the myocardium at risk of thermal damage.

## 2. Materials and Methods

### 2.1. Model Construction

**Figure 1:**
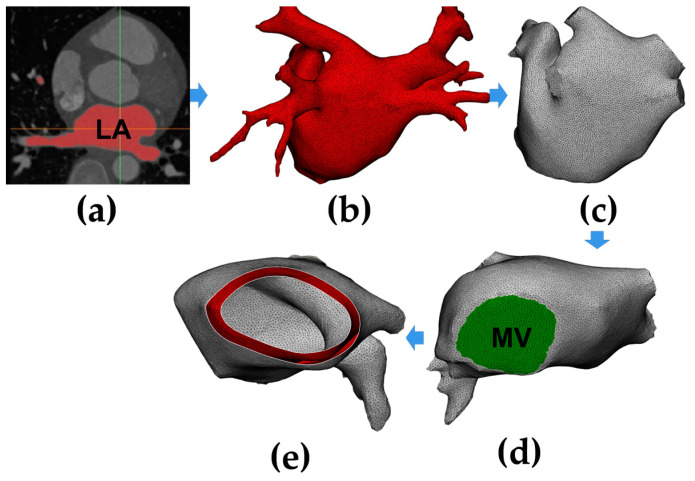
A flow chart of constructing an anatomy-based left atrium (LA) model. (**a**) Segment LA region from coronary computed tomography angiography (CTA) images; (**b**) generate the triangular mesh of the LA model; (**c**) trim the four pulmonary veins (PVs); (**d**) create the mitral valve (MV); (**e**) generate the LA model with the atrial wall.

Figure 1 shows the process of constructing an anatomy-based LA model. The process was divided into three parts: first, reconstructing the triangular mesh of the LA model with coronary computed tomography angiography (CTA) images (Figure 1a,b), then trimming the four pulmonary veins (PVs) (Figure 1c) and creating the mitral valve (MV) (Figure 1d), and finally, generating the LA model with the atrial wall (Figure 1e).

CTA images were performed using a SOMATOM Drive (Siemens, Berlin and Munich, Germany); the reconstructed image matrix size is 512 (pixel) × 512 (pixel) × 298 (slice number), the slice thickness is 0.75 mm, and the pixel size is 0.326172 mm.

The semi-automatic segmentation of the LA region from CTA images was performed using CemrgApp v2.2 (Figure 1a) [6]; the LA region was defined as the pixels contained within the LA endocardial surface, including the PVs, the MV, as well as the LA appendage (LAA) [7]. Region growing was used to separate the LA region from the selected slices. In order to reduce the impact of noise or image artefacts on the segmentation outcome, details were manually corrected (Figure 1b).

For the subsequent application of fluid dynamics boundary conditions to the model, this study trimmed the four PVs and created the MV. For PVs, all the branches except the parts that directly connected to the LA were removed, and the geometric boundaries were cut into the planes (Figure 1c); the minimum diameter, maximum diameter, and circumference of the four PVs in the model were all within the radiographic measurement range of normal human PVs [8]. For MV, due to the MV showing a similar image intensity as LA and the left ventricle, a cutting plane was manually inserted in the position of the MV; then, a circle with a diameter of 24 mm was set at the bottom of the LA model, which is within the anatomical measurement range of normal human MV (Figure 1d) [9].

Since a segmentation of the LA epicardial surface from traditional CTA images is usually not feasible due to an insufficient spatial resolution and a limited signal-to-noise ratio, in order to build the LA epicardial surface, this study assumed the thickness of the LA wall was homogeneous and the endocardial surface was dilated by 2.4 mm outward along the normal direction at each point, which is the mean measured wall thickness of the human LA (Figure 1e) [10].

### 2.2. Pulsatile Blood Flow Velocity Profiles

The pulsatile blood flow velocity profile at the MV $U_{\mathrm{MV}}$ was based on a magnetic resonance imaging (MRI) velocity record [11] and made further adjustments according to up-to-date international regulation ISO 5840-1:2021 [12]. The $U_{\mathrm{MV}}$ consists of a biphasic wave named the peak E-wave and A-wave; the definition of peak E-wave is the peak velocity of transmitral blood flow in early left ventricle diastole, and the definition of peak A-wave is peak mitral inflow velocity in late diastole due to atrial contraction [13].

**Figure 2:**
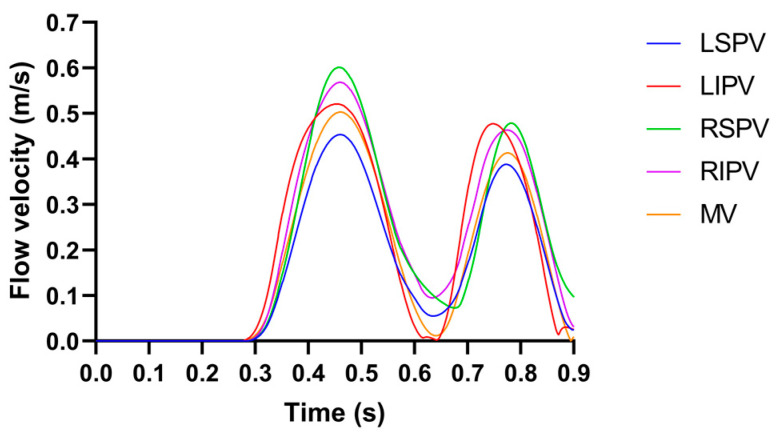
The pulsatile blood flow profiles at the four PVs and MV in the LA model, where the LSPV/LIPV/RSPV/RIPV is the left superior/left inferior/right superior/right inferior pulmonary vein.

The pulsatile blood flow profiles at the four PVs and MV in the LA model is shown in Figure 2, in which the characteristics of the pulsatile blood flow profiles at the four PVs (peak flow velocity, the ratio of peak flow velocity, and time–velocity integral, etc.) and the mean LA flow velocity are within the range described in the literature [8,14].

### 2.3. Governing Equations

This study used the AC/DC module, bio-heat transfer module, and CFD module in COMSOL Multiphysics v5.6 (COMSOL, Stockholm, Sweden) to solve the electric-thermal-fluid dynamics coupling. The electrical, thermal, and CFD equations during PFA are expressed as follows:

#### 2.3.1. Electrical Equations

The current density $J({A/m}^{2})$ and electric field intensity E(V/m) generated by PFA in LA are written as follows:

(1) J=σE

(2) E=−∇V

where V is the voltage and σ(S/m) is the electrical conductivity.

No other electric source existed inside the LA, so its potential is regulated by the Laplace equation [15]:

(3) ∇⋅(σ∇V)=0

In LA, the distributed heat source $Q({W/m}^{3})$ at a given point is proportional to the myocardial electrical conductivity and the square of the magnitude of the electric field intensity vector:

(4) $$Q=\sigma {\left|E\right|}^{2}$$

#### 2.3.2. Thermal Equations

The physical phenomena of the thermoelectric coupling problem are expressed by the Pennes biological heat transfer equation [16]:

(5) $$\rho c\frac{\partial T}{\partial t}=\nabla \cdot (k\nabla T)+Q-Q_{p}+Q_{m}-Q_{b}$$

where $\rho \left({\mathrm{kg}/m}^{3}\right)$ is the density, c(J/kg/K) is the specific heat, k(W/m/K) is the thermal conductivity, T is the temperature in K, $Q_{p}\left({W/m}^{3}\right)$ is the heat loss due to blood perfusion, and $Q_{m}\left({W/m}^{3}\right)$ is the metabolic heat generation. Since the contribution of the $Q_{p}$ and $Q_{m}$ are significantly smaller than those of other terms, they are neglected [16].

In order to include the phase change of the biological tissue due to water vaporization, the enthalpy method is included in the Pennes biological heat transfer equation (Equation (5)), as shown in the following equation [17]:

(6) $$\rho c\frac{\partial T}{\partial t}=\frac{\partial (\rho h)}{\partial t}=\frac{\partial T}{\partial t}\cdot \{\begin{array}{cc} \rho_{l}c_{l} & 0\leq T\leq 99\;{}^{\circ}C\\ H_{fg}C & 99<T\leq 100\;{}^{\circ}C\\ \rho_{g}c_{g} & T\;>\;100\;{}^{\circ}C \end{array}$$

where h is the enthalpy, $\rho_{l}$ and $c_{l}$ are the density and specific heat of myocardium before phase-change (liquid phase), and $\rho_{g}$ and $c_{g}$ are the density and specific heat of myocardium post-phase-change (gas phase), respectively. $H_{fg}\;(2.162\times 10^{9}{\;J/m}^{3})$ is the latent heat corresponding to the product of the water vaporization latent heat (2257 kJ/kg) and water density at 100 °C (958 kg/m^3^), and C (75%) is the tissue water content inside the myocardium.

#### 2.3.3. CFD Equations

The last term in Equation (5) $Q_{b}=\rho c\vec{u}\cdot \nabla T$ is the heat loss due to blood motion, where $\vec{u}(m/s)$ is the blood velocity field and is described by the incompressible Navier-Stokes equations, consisting of the momentum and mass equations, as shown in the following:

(7) $$\rho \frac{\partial \vec{u}}{\partial t}+\rho \vec{u}\cdot \nabla \cdot \vec{u}=-\nabla P+\mu \nabla^{2}\vec{u}+F$$

(8) $$\nabla \cdot \vec{u}=0$$

where P(Pa) is the pressure, $\mu \;(2.1\times 10^{-3}\;\mathrm{kg}/m/s)$ is the blood viscosity, and $F({N/m}^{-3})$ is the body forces and is neglected [18].

### 2.4. Domain

**Figure 3:**
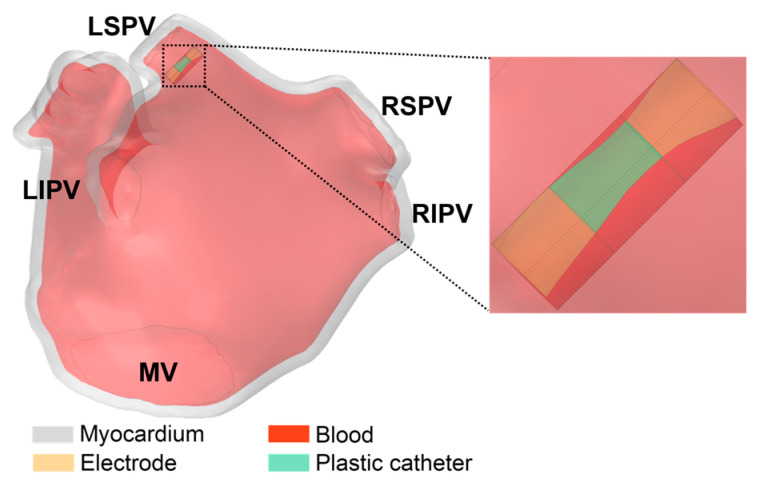
The ablation model contains the LA model and the catheter model.

Figure 3 illustrates the ablation model containing the LA model and the catheter model. The LA model consists of myocardium and blood, and the catheter model is consistent with the straight pressure catheter used in the actual PFA in animal experiments, which consists of two electrodes and is connected by an insulated plastic catheter in the middle. The diameter of the electrode and the plastic catheter is 7F, and the length of the single electrode and the plastic catheter is 2 mm and 2.5 mm, respectively.

The catheter model was placed on the left superior pulmonary vein (LSPV) ostium of the LA model, and the distance was about 6.5 mm from the LSPV ostium; this ablation target site corresponds to the circumferential pulmonary vein isolation strategy, which is a mainstream catheter ablation strategy for persistent AF. In order to simulate the effect of slight electrode pressure on the endocardium, the catheter was inserted into the endocardium at a depth of 1 mm and placed parallel to the epicardium [19].

### 2.5. Boundary Conditions

#### 2.5.1. Electrical Boundary Conditions

The monophasic pulse voltage $V_{a}=P(t)$ was applied on the left electrode, and the zero voltage $V_{b}=0$ was applied on the right electrode. The boundary of the plastic catheter and the epicardium represents the zero electric flux condition and is expressed as:

(9) n⋅σ∇V=0

where n is the normal unit vector to the epicardium surface.

- The Parameters of P(t)

**Figure 4:**
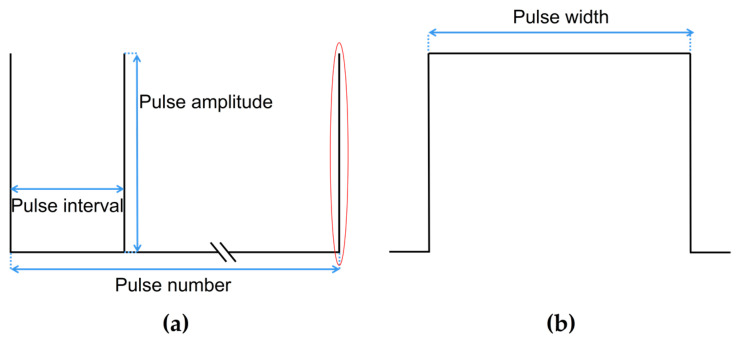
A typical monophasic PFA waveform and contained parameters, where (**a**) is the complete PFA waveform, and (**b**) is the detail of the complete waveform of a single pulse in the red.

As shown in Figure 4, a typical monophasic PFA waveform usually contains parameters such as pulse width, pulse amplitude, pulse interval, pulse number, etc. The ranges of PFA parameters used in this study were derived from the PFA in AF treatment [20] or animal experiences [21,22], as shown in the following:

The pulse width of P(t) is fixed at 100 μs as used in most studies; the pulse amplitude ranges from 1000 V to 2000 V [20]; the pulse interval ranges from 250 ms to 1000 ms [21]; and the pulse number ranges from 10 to 60 [22].

In order to highlight the influence of a single PFA parameter on the temperature rise and myocardial ablation volume of the model, this study took the remaining PFA parameters as the minimum influence on the temperature rise during PFA when investigating a single PFA parameter (minimum pulse amplitude: 1000 V, maximum pulse interval: 1000 ms, and minimum pulse number: 10). The details of the PFA parameter setting are listed in Table 1.

It can be seen from Table 1 that the influence of different PFA parameters on the temperature rise and ablation volume of the model was investigated in each of the three consecutive groups, including group 1 to 3 for pulse amplitude, group 4 to 6 for pulse interval, and group 7 to 9 for pulse number.

#### 2.5.2. Thermal Boundary Conditions

Thermal boundary conditions comprised an initial temperature boundary condition and one type of thermal boundary condition:

1. Initial temperature boundary condition: At t = 0, the initial temperature of the myocardium $T_{0}$ and the blood $T_{b}$ was set to 37 °C;
2. The second type of thermal boundary condition (constant heat flux boundary condition): The boundary of the plastic catheter and the epicardium represents the zero heat flux condition and is expressed as [17]:

(10) n⋅k∇T=0

- Methods for Simulating the Pulsatile Blood Flow

The effect of heat dissipation from intracardiac pulsatile blood flow was simulated by the CHT method and the CFD method, respectively, which have been used in previous studies, as shown in the following:

1. CHT method

The heat convection boundary condition is expressed as:

(11) $$n\cdot k\nabla T=h_{i}\left(T-T_{b}\right)$$

The corresponding thermal convective coefficient $h_{i}$, where i={m,c} was applied to the blood-myocardium and blood–catheter interfaces of the model, respectively. The $h_{m}$ and $h_{c}$ are calculated as follows [17]:

The thermal convective coefficient $h_{m}$ at the blood–myocardium interface is calculated as follows:

(12) $$u={\left(\frac{h_{e}}{h_{ref}}\right)}^{1.25}u_{ref}$$

where u (15.22 cm/s) is the mean flow velocity over a cardiac cycle at the LSPV ostium where the catheter is located, $u_{ref}\;(24\;\mathrm{cm}/s)$ is the reference flow velocity, and $h_{ref}\;(1417{\;W/m}^{2}/K)$ is the reference thermal convective coefficient obtained from $u_{ref}$.

The thermal convective coefficient $h_{c}$ at the blood–catheter interface is calculated as

(13) $$h_{c}=\frac{Nuk_{b}}{d}$$

where Nu is the Nusselt number, $k_{b}({W/m}^{2}/K)$ is the blood thermal conductivity, and d(m) is the diameter of the catheter. The Nu can be approximated as follows:

(14) Nu=0.683Re0.466Pr0.333

where Re and Pr are the Reynolds and Prandtl numbers, calculated using Equations (15) and (16), respectively.

(15) Re=ρbu→dμ

(16) $$\mathrm{Pr}=\frac{c_{b}\mu }{k_{b}}$$

where $\rho_{b}\left({\mathrm{kg}/m}^{3}\right)$ is the blood density and $c_{b}(J/\mathrm{kg}/K)$ is the blood specific heat. Finally, this study obtained $h_{e}=984\;({W/m}^{2}/K)$ and $h_{c}=4372\;({W/m}^{2}/K)$, respectively.

2. CFD Method

A no-slip condition was applied to the blood–myocardium interface and blood–catheter interface. The four PVs were set as inflows with the condition of 10 mmHg [23], and the MV was set as the outflow with the pulsatile blood flow velocity profile $U_{\mathrm{MV}}$ (see Section 2.2).

The fluid type in LA was determined by calculating the Reynolds number:

(17) Re=ρu→Dμ

where D(m) is the diameter of the PVs or MV. As the Reynolds number for the peak blood flow velocities in the PVs and MV are all less than 2000, the fluid type in LA was set as laminar flow [24].

**Figure 5:**
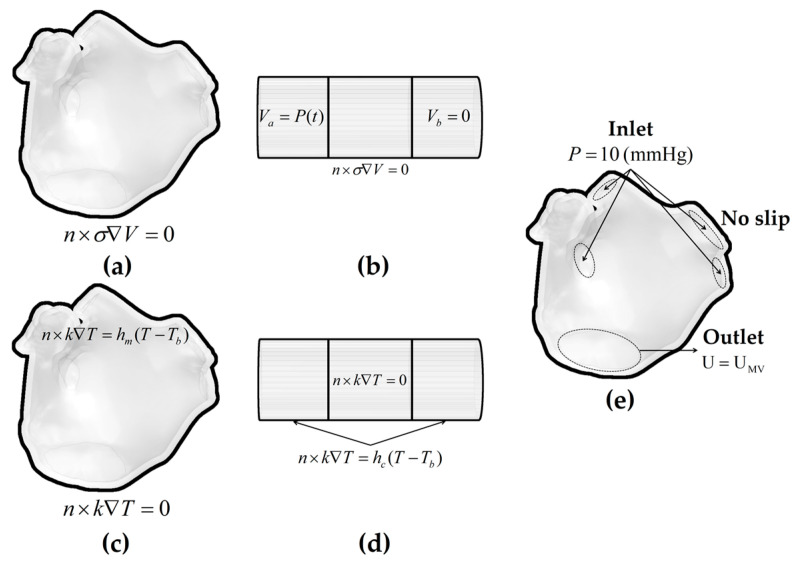
The electrical boundary conditions, thermal boundary conditions, and fluid dynamics boundary conditions of the ablation model. Electrical boundary conditions of (**a**) the LA model and (**b**) the catheter model; thermal boundary conditions of (**c**) the LA model and (**d**) the catheter model in the CHT method; and fluid dynamics boundary conditions of (**e**) the LA model in the CFD method.

Figure 5 illustrates the boundary conditions of the ablation model.

### 2.6. Material Properties

The electrical and thermal properties of the ablation model are listed in Table 2.

The myocardial electrical conductivity is considered electric field intensity-dependent and temperature-dependent. First, this study used a sigmoid function that relates the myocardial electrical conductivity to the electric field intensity magnitude during PFA and is expressed as [25]:

(18) $$\sigma (E)=\sigma_{0}+\frac{\sigma_{1}-\sigma_{0}}{1+10e^{{}^{-}\frac{\left(|E|-58000\right)}{3000}}}$$

where $\sigma_{0}$ and $\sigma_{1}$ are the pre- and post-electroporation myocardial electrical conductivities, respectively. Since no specific data are currently available for myocardium, this study chose the electrical conductivity values at 10 Hz and 500 kHz to simulate cardiac cells pre- and post-electroporation, i.e., $\sigma_{0}=0.0537\;S/m$ and $\sigma_{1}=0.281\;S/m$, respectively [25,26]. Second, the myocardial electrical conductivity is considered an exponential growth of 1.5%/°C based on σ(E) up to 100 °C, and then it linearly decreases from 100 °C to 105 °C to simulate the myocardium desiccation process [3,17].

(19) $$\sigma (E,T)=\{\begin{array}{ll} \sigma (E)e^{0.015(T-37)} & 37\;{}^{\circ}C<T\leq 100\;{}^{\circ}C\\ 1.371-0.274\left(T-100\right) & 100\;{}^{\circ}C<T\leq 105\;{}^{\circ}C\\ 1.371\times 10^{-4} & T\;>\;105\;{}^{\circ}C \end{array}$$

The myocardial thermal conductivity is considered temperature-dependent and grows linearly 0.12%/°C from 0.531 W/m/K (the value of the myocardial thermal conductivity assessed at 37 °C) up to 100 °C, then it is kept constant [17].

(20) $$k(T)=\{\begin{array}{ll} 0.531+0.0012(T-37) & 0<T\leq 100\;{}^{\circ}C\\ 0.606 & T\;>\;100\;{}^{\circ}C \end{array}$$

### 2.7. Data Computation and Criterion

#### 2.7.1. Data Computation

The ablation model used in this study was discretized using tetrahedral mesh elements and further refined the area where the catheter is in contact with the myocardium since this location expected a higher electrical and thermal gradient. A mesh sensitivity analysis was conducted to compute the optimal number of mesh elements by progressively refining the mesh until the absolute difference of the maximum temperature of myocardium and blood, and the maximum velocity of pulsatile blood flow at the four PVs and MV during PFA are less than 0.5% compared to the previous mesh size [5]. Finally, the minimum and maximum lengths of the element in the ablation model are 0.4 mm and 1 mm, respectively.

The application of the CFD method led to a model based on the electric-thermal-fluid dynamics coupling, and the number of tetrahedral elements of the ablation model used in this study exceeded a million. Therefore, this study used the supercomputer (Tianhe-I, Tianjin, China) to solve this problem with a tremendous amount of computation, which contains two Intel Xeon Gold 5218 with 32 cores in total and 128 GB of RAM, and a used multifrontal massively parallel sparse direct solver (MUMPS) and segregated solution approach, in which the MUMPS performs the factorization of linear systems in the form of Ax=b, where the matrix A is a square sparse matrix and is factorized to determine the solution x [27,28].

#### 2.7.2. Myocardial Ablation Volume Criterion

At higher PFA doses, the mechanism of myocardial ablation caused by PFA changes from IRE alone to IRE combined with hyperthermia; this study used different criteria to determine the myocardial ablation volume caused by the above two mechanisms.

For IRE, presently, a specific electric field intensity threshold is usually used to estimate whether PFA causes effective myocardial injury; this study used the IRE threshold for cardiac cells, defined as the electric field intensity exceeding 1000 V/cm [29].

Two criteria were used to determine the myocardial ablation volume caused by hyperthermia. First, this study used the temperature isosurface criterion, defined as the temperature exceeding 50 °C, which corresponds to a reasonable estimate of the irreversible myocardial injury caused by hyperthermia [30].

However, Mario et al. [3] considered that since the pulses in PFA are very intense but have a very short duration, the temperature isosurface criterion may not reflect the realistic thermal damage area. Hence, this study secondly used the Arrhenius equation, which associates cell activity with exposure time and temperature through a first-order kinetics process and is regarded as a much more accurate estimator of thermal damage for short exposure durations. The probability of tissue damage can be predicted using $P=(1-e^{-\Omega (t)})\cdot 100\%$, where Ω(t) is calculated as follows:

(21) $$\Omega (t)=\int_{0}^{t}Ae^{-\frac{\Delta E}{RT(\tau )}}d\tau$$

where t(s) is the heating time, $A\;(2.94\times 10^{39}{\;s}^{-1})$ is the frequency factor, $\Delta E\;(2.596\times 10^{5}\;J/\mathrm{mol})$ is the activation energy, and R (8.314 J/mol/K) is the universal gas constant [31]. Irreversible myocardial injury is considered for a probability threshold greater than 63% (Ω(t)=1).

#### 2.7.3. Difference Criterion

In this study, the size of the myocardial ablation volume is in cubic mm, and the difference in the size of the myocardial ablation volume between the CHT method and CFD method is insignificant for values less than 1 mm^3^ since this value is approximate to the deviation (±0.5 mm) observed in experimental studies [5]. Likewise, the differences in the maximum temperature of myocardium and blood between the CHT method and CFD method are considered insignificant for values less than 1 °C since this value is the system security (±0.5 °C) of the temperature fiber optic sensor used to measure the temperature rise during PFA [32].

## 3. Results

### 3.1. The Velocity Characteristics of Pulsatile Blood

**Figure 6:**
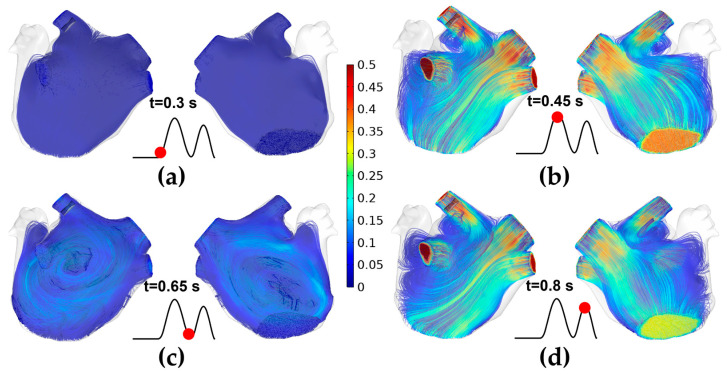
The velocity streamlines of pulsatile blood in the ablation model at four moments during a single cardiac cycle. (**a**) t = 0.3 s; (**b**) t = 0.45 s; (**c**) t = 0.65 s; (**d**) t = 0.8 s.

Figure 6 shows the velocity streamlines of pulsatile blood in the ablation model at four moments during a single cardiac cycle. Specifically, at t = 0.3 s, the MV is in the initial state from close to open, and almost no blood flows out of the MV, resulting in the average flow velocity in the LA being minimal ($\bar{v}$ = 0.005 m/s). At t = 0.45 s, the MV opens and the left ventricle rapidly fills, resulting in the average flow velocity ($\bar{v}$ = 0.224 m/s) reaching the maximum during a single cardiac cycle; at the moment, part of the blood flow at the LIPV ostium is diverted when it touches the LAA and forms a counterclockwise vortex between LSPV and LIPV. At t = 0.65 s, it is under the diastasis phase, during which no significant blood flow across the MV ($\bar{v}$ = 0.087 m/s), the blood flow from the four PVs converges in the middle of the LA and forms a clockwise vortex. At t = 0.8 s, the LA contracts, and the velocity characteristic is similar to those at t = 0.45 s, but the average flow velocity ($\bar{v}$ = 0.076 m/s) is lower than the former.

### 3.2. The Maximum Temperature of Myocardium and Blood

**Figure 7:**
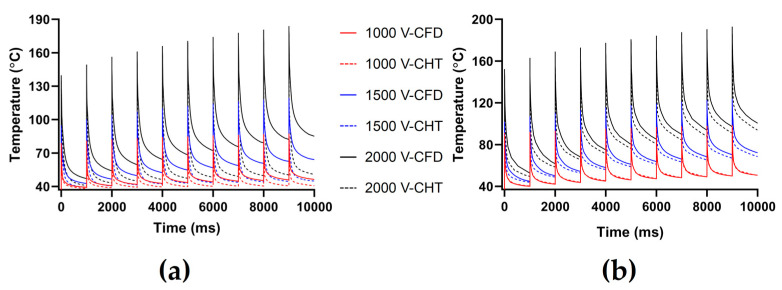
The maximum temperature curves of (**a**) myocardium and (**b**) blood during PFA under different pulse amplitudes.

**Figure 8:**
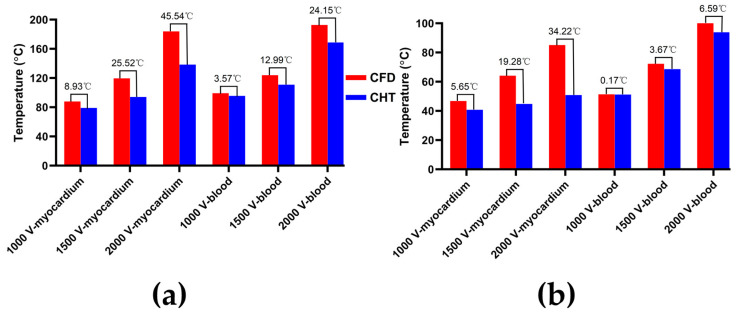
The statistical results of the maximum temperature curves of myocardium and blood at (**a**) the end of the last pulse and (**b**) the end of the last pulse interval under different pulse amplitudes, where the numbers between the top of the bars in the figures represent the temperature difference between the CHT method and the CFD method.

**Figure 9:**
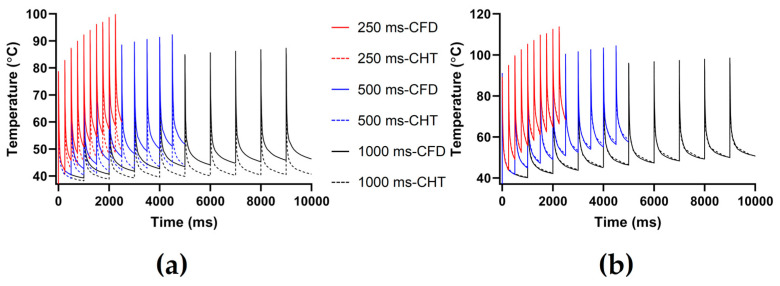
The maximum temperature curves of (**a**) myocardium and (**b**) blood during PFA under different pulse intervals.

**Figure 10:**
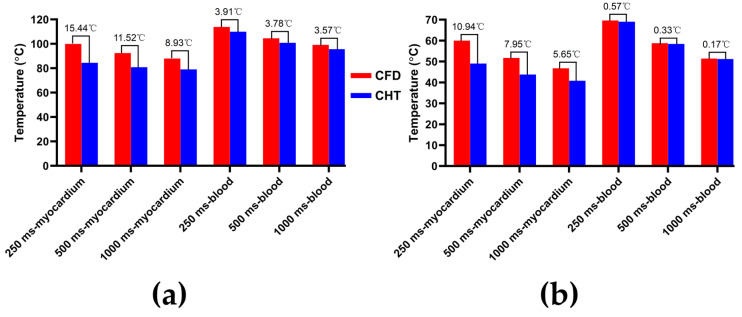
The statistical results of the maximum temperature curves of myocardium and blood at (**a**) the end of the last pulse and (**b**) the end of the last pulse interval under different pulse intervals.

**Figure 11:**
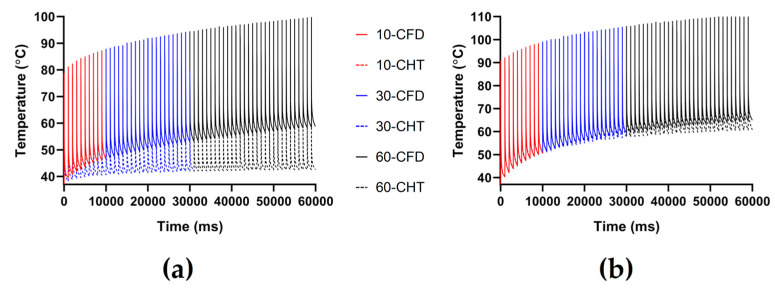
The maximum temperature curves of (**a**) myocardium and (**b**) blood during PFA under different pulse numbers.

**Figure 12:**
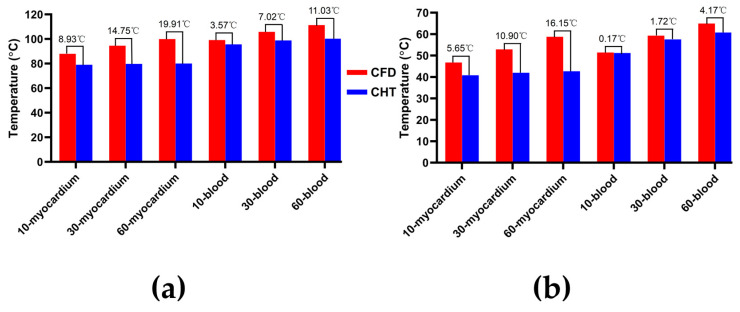
The statistical results of the maximum temperature curves of myocardium and blood at (**a**) the end of the last pulse and (**b**) the end of the last pulse interval under different pulse numbers.

Figures 7, 9 and 11 show the maximum temperature curves of myocardium and blood during PFA under different PFA parameters. Figures 8, 10, and 12 show the statistical results of the maximum temperature curves of myocardium and blood at the end of the last pulse and the end of the last pulse interval under different PFA parameters.

In general, under different PFA parameters, compared with the CFD method, the CHT method underestimated the maximum temperature of myocardium and blood during PFA. The differences in the maximum temperature of myocardium and blood between the two methods at the end of the last pulse are significant (>1 °C), and the differences in the maximum temperature of blood at the end of the last pulse interval are significant (>1 °C) only at pulse amplitude greater than 1000 V or pulse number greater than 10. 

#### 3.2.1. Influence of Different Pulse Amplitudes

The PFA parameters used here are the pulse interval of 1000 ms, the pulse number of 10, and the pulse amplitude of 1000 V, 1500 V, and 2000 V, respectively. According to Figure 7 and Figure 8, under the same heat dissipation method and different pulse amplitudes, the differences between the CHT method and the CFD method at the end of the last pulse and the end of the last pulse interval in the maximum temperature of myocardium and blood are positively correlated with the pulse amplitude (Pearson correlation coefficient *r* > 0.90).

According to Figure 8a, under the same pulse amplitude, the differences between the two methods in the maximum temperature of myocardium and blood at the end of the last pulse are significant (>1 °C). Under the pulse amplitude of 2000 V, the differences reach the maximum of 45.54 °C (myocardium) and 24.15 °C (blood) at the end of the last pulse, respectively.

According to Figure 8b, under the same pulse number, the difference between the two methods in the maximum temperature of blood at the end of the last pulse interval is insignificant (<1 °C) only under the pulse amplitude of 1000 V. Under the pulse amplitude of 2000 V, the differences reach the maximum of 34.22 °C (myocardium) and 6.59 °C (blood) at the end of the last pulse interval, respectively.

#### 3.2.2. Influence of Different Pulse Intervals

The PFA parameters used here are the pulse amplitude of 1000 V, the pulse number of 10, and the pulse interval of 250 ms, 500 ms, and 1000 ms, respectively. According to Figure 9 and Figure 10, under the same heat dissipation method and different pulse intervals, the differences between the CHT method and the CFD method at the end of the last pulse and the end of the last pulse interval in the maximum temperature of myocardium and blood are negatively correlated with the pulse interval (*r* < −0.95).

According to Figure 10a, under the same pulse interval, the differences between the two methods in the maximum temperature of myocardium and blood at the end of the last pulse are significant (>1 °C). Under the pulse interval of 250 ms, the differences reach the maximum of 15.44 °C (myocardium) and 3.91 °C (blood) at the end of the last pulse, respectively.

According to Figure 10b, under the same pulse interval, the differences between the two methods in the maximum temperature of myocardium at the end of the last pulse interval are significant (>1 °C), and the differences of blood at the end of the last pulse interval are insignificant (<1 °C). Under the pulse interval of 250 ms, the differences reach the maximum of 10.94 °C (myocardium) and 0.57 °C (blood) at the end of the last pulse interval, respectively.

#### 3.2.3. Influence of Different Pulse Numbers

The PFA parameters used here are the pulse amplitude of 1000 V, the pulse interval of 250 ms, and the pulse number of 10, 30, and 60, respectively. According to Figure 11 and Figure 12, under the same heat dissipation method and different pulse numbers, the differences between the CHT method and the CFD method at the end of the last pulse and the end of the last pulse interval in the maximum temperature of myocardium and blood are positively correlated with the pulse number (*r* > 0.94).

According to Figure 12a, under the same pulse number, the differences between the two methods in the maximum temperature of myocardium and blood at the end of the last pulse are significant (>1 °C). Under the pulse number of 60, the differences reach the maximum of 19.91 °C (myocardium) and 11.03 °C (blood) at the end of the last pulse, respectively.

According to Figure 12b, under the same pulse number, the difference between the two methods in the maximum temperature of blood at the end of the last pulse interval is insignificant (<1 °C) only under the pulse number of 10. Under the pulse number of 60, the differences reach the maximum of 16.15 °C (myocardium) and 4.17 °C (blood) at the end of the last pulse interval, respectively.

### 3.3. Myocardial Ablation Volume

**Figure 13:**
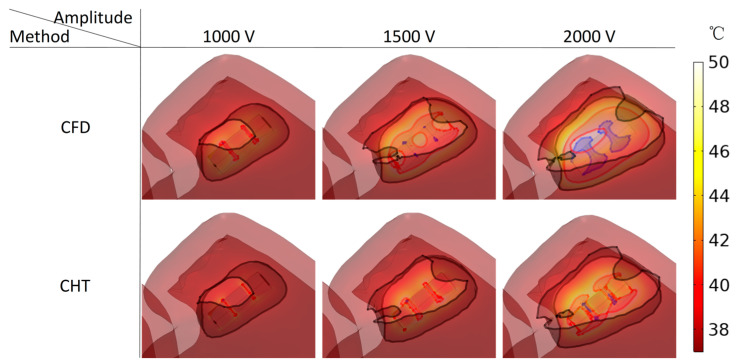
Three kinds of myocardial ablation isosurfaces at the end of the last pulse under different pulse amplitudes, in which the black area represents the electric field intensity isosurface (E=1000 V/cm), representing the myocardial ablation volume caused by the IRE mechanism; the red area represents the temperature ablation isosurface (T=50 °C ), and the blue area represents the Arrhenius equation ablation isosurface (Ω(t)=1), both representing the myocardial ablation volume caused by the hyperthermia mechanism.

**Figure 14:**
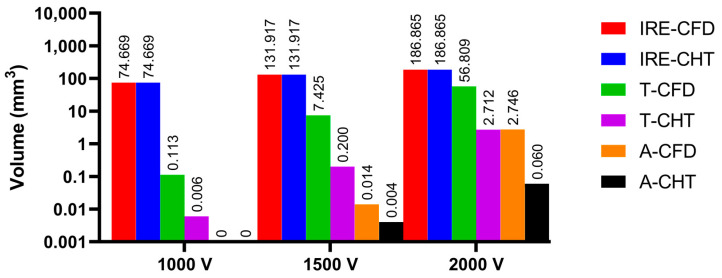
The statistical results of the volume sizes of the three kinds of myocardial ablation isosurfaces under different pulse amplitudes, where the numbers on the top of the bars in the figure represent the volume size. In the right legends, IRE-CFD and IRE-CHT represent the IRE ablation volume under the CFD method and the CHT method, respectively; T-CFD and T-CHT represent the temperature ablation volume under the CFD method and the CHT method, respectively; and A-CFD and A-CHT represent the Arrhenius equation volume under the CFD method and the CHT method, respectively.

**Figure 15:**
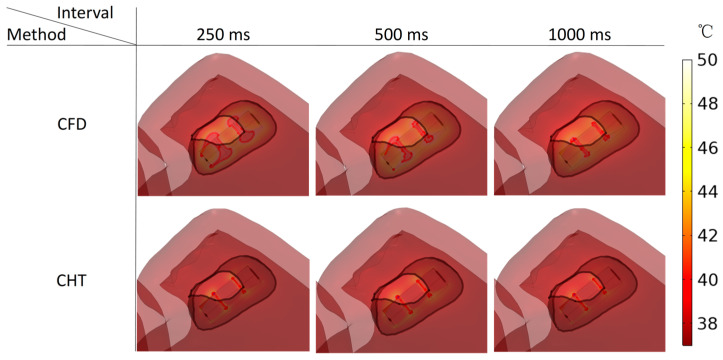
Three kinds of myocardial ablation isosurfaces at the end of the last pulse under different pulse intervals.

**Figure 16:**
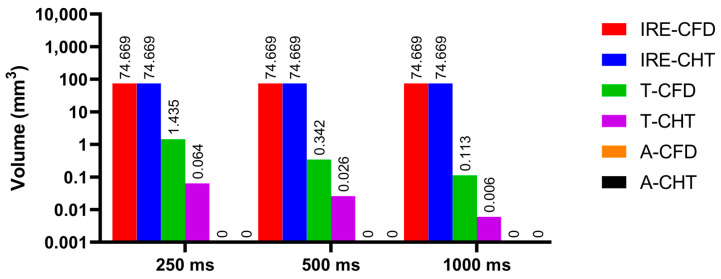
The statistical results of the volume sizes of the three kinds of myocardial ablation isosurfaces under different pulse intervals.

**Figure 17:**
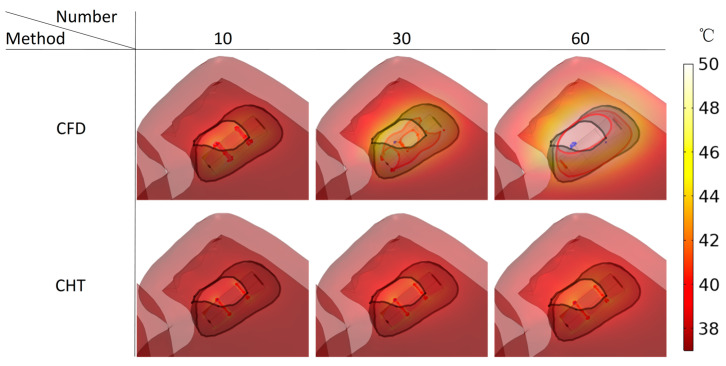
Three kinds of myocardial ablation isosurfaces at the end of the last pulse under different pulse numbers.

**Figure 18:**
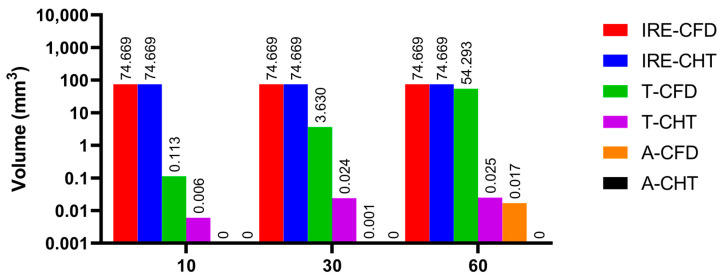
The statistical results of the volume sizes of the three kinds of myocardial ablation isosurfaces under different pulse numbers.

Figures 13, 15, and 17 show three kinds of myocardial ablation isosurfaces at the end of the last pulse under different PFA parameters, in which the black area represents the electric field intensity isosurface (E=1000 V/cm), representing the myocardial ablation volume caused by the IRE mechanism (hereinafter referred to as IRE ablation isosurface); the red area represents the temperature ablation isosurface (T=50 °C); and the blue area represents the Arrhenius equation ablation isosurface (Ω(t)=1), both representing the myocardial ablation volume caused by the hyperthermia mechanism. Figures 14, 16, and 18 show the statistical results of the volume sizes of the three kinds of myocardial ablation isosurfaces (hereinafter referred to as IRE, temperature, and Arrhenius equation ablation volume, respectively) under different PFA parameters.

In general, due to the high electrical conductivity of the electrodes and the insulation of the plastic catheter, and the electrical conductivity of the myocardium is quite different from that of the catheter, the areas where the electrodes are in contact with the plastic catheter and the catheter is in contact with the myocardium have a higher electric field intensity, according to the Equation (4) that as a result, these areas have a higher distributed heat source. Finally, under different PFA parameters, the hyperthermia ablation isosurfaces (containing temperature ablation isosurface and Arrhenius equation ablation isosurface) are concentrated in these areas.

Since the IRE ablation volume is determined by a specific electric field intensity threshold and is only related to the pulse amplitude, under the same pulse amplitude and different heat dissipation methods, the IRE ablation volumes are the same. Meanwhile, compared with the CFD method, the CHT method underestimates the hyperthermia ablation volume (containing temperature ablation volume and Arrhenius equation ablation volume); the differences between the two methods are significant (>1 mm^3^) only at pulse amplitude greater than 1000 V, the pulse interval of 250 ms, or the pulse number greater than 10. Additionally, under different PFA parameters, the hyperthermia ablation isosurfaces are completely wrapped by the IRE ablation isosurfaces in the myocardium. 

#### 3.3.1. Influence of Different Pulse Amplitudes

According to Figure 13 and Figure 14, under the same heat dissipation method and different pulse amplitudes, the IRE ablation volumes are positively correlated with the pulse amplitude (*r* > 0.99).

Under the pulse amplitude of 1000 V, although there are temperature ablation isosurfaces in the areas where the electrodes are in contact with the plastic catheter under the CFD method and the CHT method, the volumes are all less than 1 mm^3^. Meanwhile, there is no Arrhenius equation ablation isosurface under the two methods.

Under the pulse amplitude of 1500 V and the CFD method, the temperature ablation isosurface covers part of the area where the catheter is in contact with the myocardium. Under the CHT method, the temperature ablation isosurfaces in the area where the electrodes are in contact with the plastic catheter further increase, but the volumes are still less than 1 mm^3^. Although the areas where the electrodes are in contact with the plastic catheter appear to be Arrhenius equation ablation isosurfaces under the two methods, the volumes are less than 0.1 mm^3^.

Under the pulse amplitude of 2000 V and the CFD method, the temperature ablation isosurface completely covers the area where the catheter is in contact with the myocardium. The areas where the electrodes are in contact with the plastic catheter appear additional with the Arrhenius equation ablation isosurface, and the volumes reach 2.712 mm^3^. Under the CHT method, the areas where the catheter is in contact with the myocardium appear with additional temperature ablation isosurfaces. Although the Arrhenius equation ablation isosurfaces in the area where the electrodes are in contact with the plastic catheter further increase, the volumes are less than 0.1 mm^3^.

Under the pulse amplitude greater than 1000 V, the differences between the CHT method and the CFD method in the temperature ablation volume are significant (>1 mm^3^), and under the pulse amplitude of 2000 V, the difference between the two methods in the Arrhenius equation ablation volume is significant (>1 mm^3^).

#### 3.3.2. Influence of Different Pulse Intervals

According to Figure 15 and Figure 16, under the pulse interval of 250 ms and the CFD method, there are temperature ablation isosurfaces with volume sizes greater than 1 mm^3^ in the areas where the electrodes are in contact with the plastic catheter and the catheter is in contact with the myocardium. In the other conditions, although there are temperature ablation isosurfaces in the area where the electrodes are in contact with the plastic catheter, the volumes are all less than 1 mm^3^. Meanwhile, there is no Arrhenius equation ablation isosurface under the two methods.

Under the pulse interval of 250 ms, the difference between the CHT method and the CFD method in the temperature ablation volume is significant (>1 mm^3^).

#### 3.3.3. Influence of Different Pulse Numbers

According to Figure 17 and Figure 18, under the different pulse numbers and the CHT method, although there are temperature ablation isosurfaces in the areas where the electrodes are in contact with the plastic catheter, the volumes are all less than 0.1 mm^3^. Meanwhile, there is no Arrhenius equation ablation isosurface under the CHT method.

Under the pulse number of 10 and the CFD method, although there are temperature ablation isosurfaces in the areas where the electrodes are in contact with the plastic catheter, the volumes are less than 1 mm^3^. Under the pulse number of 30 and the CFD method, the areas where the catheter is in contact with the myocardium appear with additional temperature ablation isosurfaces. Although the Arrhenius ablation isosurface appears in the areas where the catheter is in contact with the myocardium, the volume is only 0.001 mm^3^. Under the pulse number of 60 and the CFD method, the temperature ablation isosurface completely covers the area where the catheter is in contact with the myocardium. Although the Arrhenius equation ablation isosurface in the area where the catheter is in contact with the myocardium further increases, the volume is less than 0.1 mm^3^.

Under the pulse number greater than 10, the differences between the CHT method and the CFD method in the temperature ablation volume are significant (>1 mm^3^).

## 4. Discussion

### 4.1. Influence of Different Heat Dissipation Methods

This study constructed an anatomy-based LA model and compared the effect of heat dissipation between the CHT method and the CFD method in simulating the pulsatile blood flow during PFA. The temperature rise within the myocardium during PFA is a complex function of joule heating and several other competing heat transfer mechanisms. The primary heat transfer mechanisms are conduction heat transfer from the electrode to the myocardium and convection heat transfer from the myocardium and from the electrode to the pulsatile blood flow. In current relevant studies, Pennes biological heat transfer equation Equation (5) has been used to simulate conduction heat transfer in a relatively simple and accurate way; however, the simulation method of convective heat transfer is still under investigation.

As stated in the introduction, in the computational study, using the CHT method can avoid the significant increase in the model construction process and the computational complexity brought by the CFD method. However, the CHT method currently has two significant problems: First, in current relevant studies, the thermal convective coefficients of blood-myocardium and blood–catheter interface are usually derived from the average thermal convective coefficients, which are derived from an approximate estimate [33] or calculated based on the intracardiac blood flow velocity [17]; additionally, the average thermal convective coefficient is only valid when the myocardial surface is isothermal, but the myocardial surface is apparently not isothermal during PFA [34]. Second, the selection of the thermal convective coefficient highly affects the maximum temperature of myocardium and blood during PFA under the CHT method, and it seems to cause the CHT method cannot accurately simulate the maximum temperature of myocardium and blood under the CFD method simultaneously.

In detail, this study based on u (15.22 cm/s) obtained $h_{e}=984\;({W/m}^{2}\cdot K)$ and $h_{c}=4372\;({W/m}^{2}\cdot K)$, respectively. Compared with the CFD method, the CHT method based on these two thermal convective coefficients significantly underestimated the differences in the maximum temperature of myocardium but did not significantly underestimate the differences in the maximum temperature of blood except at certain PFA parameters. In contrast, Ana et al. [5] based on u (8.5 cm/s) obtained $h_{e}=610\;({W/m}^{2}\cdot K)$ and $h_{c}=3346\;({W/m}^{2}\cdot K)$, respectively. Compared with the CFD method, the CHT method based on these two thermal convective coefficients lower than that used in this study can predict the maximum temperature of myocardium well (difference < 3 °C) but drastically overestimates the maximum temperature of blood (difference > 15 °C).

**Figure 19:**
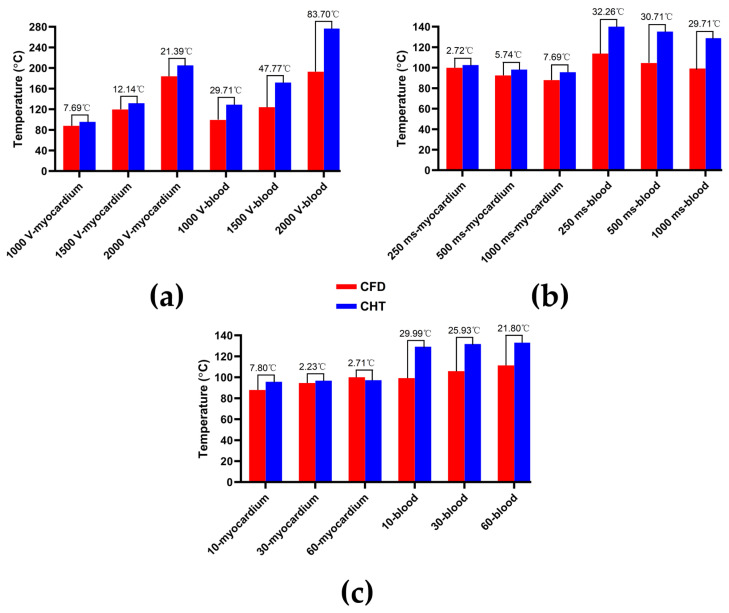
Based on the thermal convective coefficients in [5,17] obtained the statistical results of the maximum temperature curves of myocardium and blood at the end of the last pulse under different PFA parameters. (**a**) Under different pulse amplitudes; (**b**) under different pulse intervals; (**c**) under different pulse numbers.

The reason for the above phenomenon is that the thermal convective coefficients in this study and in [5,17] are different. In order to prove this point, this study based on the thermal convective coefficients in [5,17] obtained the statistical results of the maximum temperature curves of myocardium and blood at the end of the last pulse under different PFA parameters. As shown in Figure 19, first, compared with the CFD method, the CHT method overestimated the maximum temperature of blood at the end of the last pulse under different PFA parameters, which is consistent with the results in [5,17]. Second, under the different PFA parameters, the differences in the maximum temperature of the myocardium between the two methods are smaller than the results in Section 3.2 by an average of 60.21% (different pulse amplitudes), 51.18% (different pulse intervals), and 29.62% (different pulse numbers), respectively; in contrast, the differences in the maximum temperature of blood between the two methods are larger than the results in Section 3.2 by an average of 516% (different pulse amplitudes), 823% (different pulse intervals), and 469% (different pulse numbers), respectively, which is also consistent with the results in [5,17], i.e., the differences in the maximum temperature of blood between the two methods are larger than the differences in the maximum temperature of the myocardium.

In general, modifying the above two thermal convective coefficients will make the maximum temperature curves of myocardium and blood under the CHT method simultaneously move down (increase thermal convective coefficient) or up (decrease thermal convective coefficient) along the direction of the y-axis representing temperature relative to the maximum temperature curves of myocardium and blood under the CFD method. Therefore, the CHT method based on different thermal convective coefficients seems to accurately simulate only one of the maximum temperatures of myocardium or blood under the CFD method, but the other is consistently underestimated or overestimated.

### 4.2. Temperature Rise and Myocardial Ablation Volume during PFA

This study compared the influence of different PFA parameters on the maximum temperature of myocardium and blood and the volume sizes of the three types of myocardial ablation volumes. Since the maximum temperature of myocardium and blood during PFA depends on the temperature rise caused by each pulse and the temperature decrease during each pulse interval, the pulse amplitude and pulse interval are positively and negatively correlated with the maximum temperature of the myocardium and blood, as well as the size of the hyperthermia ablation volume, respectively. The pulse number influences the accumulation of the temperature rise caused by each pulse minus the temperature decrease during each pulse interval, so it is positively correlated with the maximum temperature of myocardium and blood, as well as the size of the hyperthermia ablation volume.

Although within the range of the PFA parameters used in this study, the hyperthermia ablation isosurfaces are completely wrapped by the IRE ablation isosurfaces in the myocardium; this means that the temperature rise during PFA may not lead to the appearance of additional hyperthermia ablation area beyond the IRE ablation area in the myocardium. However, at higher PFA doses than those used in this study, the temperature rise in the myocardium during PFA may lead to the appearance of additional hyperthermia ablation area beyond the IRE ablation area or further beyond the myocardium, thereby putting the tissue adjacent to the myocardium (such as PVs, esophagus, phrenic nerve, etc.) at risk of thermal damage.

Meanwhile, although the temperature distribution in the myocardium decreased rapidly with the distance between the myocardium and the catheter, at certain PFA parameters in this study, the highest temperature in the area where the catheter contacted the myocardium and the blood exceeded 100 °C and 70 °C, respectively; however, it is unclear whether the temperature rise in such a short duration is likely to lead to the occurrence of steam pops and the formation of soft thrombi during PFA. For security, Mario et al. [3] considered that irrigated catheters to avoid excessive heating near the electrodes could be helpful for PFA in AF treatment, and it is critical for physicians to be mindful of PFA parameters to maintain the non-thermal effects of PFA and prevent unnecessary damage to surrounding healthy tissues during PFA [35].

### 4.3. Compared with Experimental Results in Other Studies

This study compared the results with experimental results in other recent studies, which support the reliability of the computational results in this study to a certain extent.

Tomás et al. [32] applied PFA on the epicardium of a rat, and the record of the optical temperature probe located at the point of contact between the electrode and the tissue showed that the temperature sharply increased during each pulse and subsequently slowly dissipated by conduction between consecutive pulses. The characteristics of the maximum temperature curve of myocardium during PFA in this study are consistent with the above.

Guido et al. [36] applied PFA on the LA of swine and used PFA parameters similar to those used in this study (pulse amplitude of 900 V, internal frequency of 100 kHz, pulse interval of about 850 ms, and pulse number of 60), the results showed no temperature rise in the esophageal probe during PFA. The results of this study are consistent with the above, suggesting that the temperature rise during PFA may not lead to the appearance of additional hyperthermia ablation areas beyond the myocardium.

### 4.4. Limitations

The first limitation is that the results and conclusions in this study are based on specific myocardial electrical conductivity and electric field intensity damage threshold. However, to date, the myocardial electrical conductivity is not well characterized for low frequencies (as is the case for PFA) since they are based on estimations of those obtained experimentally above 1 MHz [37]. Meanwhile, the IRE threshold for cardiac cells varies with PFA parameters within a specific range [38].

The second limitation is that this study did not simulate the pulsatile blood flow induced by real LA contraction and movement during a single cardiac cycle; instead, the LA wall is treated as a rigid wall, and fluid boundary conditions are applied to simulate the pulsatile blood flow. It represents a major approximation, as the introduction of a moving LA wall is likely to affect the blood flow in the LA by increasing the swirling motion [39]. But this limitation may not have affected the results of this study, since this study only investigated the situation when the ablation target site is located at the LSPV ostium during PFA and did not involve the ablation target site in other parts of LA; the characteristic of pulsatile blood flow at the LSPV ostium in this study is within the range described in the literature.

## Figures and Tables

**Table 1:** The details of the PFA parameter setting.

Group	Pulse Amplitude/V	Pulse Interval/ms	Pulse Number
1	1000	1000	10
2	1500	1000	10
3	2000	1000	10
4	1000	1000	10
5	1000	250	10
6	1000	500	10
7	1000	1000	10
8	1000	1000	30
9	1000	1000	60

**Table 2:** The electrical and thermal properties of the ablation model [17,25,26].

Element/Material		$\rho ({\mathrm{kg}/m}^{3})$	c(J/kg/K)	k(W/m/K)	σ(S/m)
Electrode		21500	132	71	4.6 × 10^6^
Plastic Catheter		70	1045	0.026	1 × 10^−5^
Blood		1000	4180	0.541	0.667
Myocardium	Liquid phase	1060	3111	0.531	0.0537 */0.281 **
	Gas phase	370.44	2155.92		

* Pre-electroporation myocardial electrical conductivity; ** Post-electroporation myocardial electrical conductivity.

## Data Availability

The datasets used and/or analyzed during the current study are available from the corresponding author on reasonable request.
